# Bottom-Up Construction of Complex Biomolecular Systems With Cell-Free Synthetic Biology

**DOI:** 10.3389/fbioe.2020.00213

**Published:** 2020-03-24

**Authors:** Nadanai Laohakunakorn, Laura Grasemann, Barbora Lavickova, Grégoire Michielin, Amir Shahein, Zoe Swank, Sebastian J. Maerkl

**Affiliations:** ^1^School of Biological Sciences, Institute of Quantitative Biology, Biochemistry, and Biotechnology, University of Edinburgh, Edinburgh, United Kingdom; ^2^School of Engineering, Institute of Bioengineering, École Polytechnique Fédérale de Lausanne, Lausanne, Switzerland

**Keywords:** cell-free synthetic biology, cell-free protein synthesis, *in vitro* reconstitution, microfluidics, compartmentalization, artificial cell, *in vitro* replication

## Abstract

Cell-free systems offer a promising approach to engineer biology since their open nature allows for well-controlled and characterized reaction conditions. In this review, we discuss the history and recent developments in engineering recombinant and crude extract systems, as well as breakthroughs in enabling technologies, that have facilitated increased throughput, compartmentalization, and spatial control of cell-free protein synthesis reactions. Combined with a deeper understanding of the cell-free systems themselves, these advances improve our ability to address a range of scientific questions. By mastering control of the cell-free platform, we will be in a position to construct increasingly complex biomolecular systems, and approach natural biological complexity in a bottom-up manner.

## 1. Introduction

Synthetic biology promises to transform diverse domains including biomanufacturing, healthcare, food production, sustainable energy, and environmental remediation, by applying engineering principles to the design and construction of biological systems (Endy, 2005). Specifically, this was stipulated to involve abstracting away intricate biological complexity into simpler parts and modules whose behavior can be quantified (Heinemann and Panke, 2006; Arkin, 2008; Canton et al., 2008). The process of “building” thus involves assembling these subsystems together to obtain a required function, while quantitatively characterized components and their interactions ensure that the overall system may be predictively designed.

Practice currently diverges from the ideal framework set out above, due to the fact that we do not yet have a reliable approach to managing biological complexity (Kwok, 2010). While the idea of abstracting the behavior of a biological process, such as gene expression, into a simple mathematical model may indeed work well for single genes in isolation, as the gene circuit increases in size and complexity, the increased enzymatic and metabolic burden leads to reduced gene expression, changes in host cell state and growth rate, and increasing negative selection pressure. A seemingly modular component naturally loses its modularity as the system becomes more complex, and thus a major bottleneck preventing the current practice of synthetic biology from attaining the ideals outlined above lies in the transition from simple parts and circuits to larger systems (Purnick and Weiss, 2009).

There are several approaches to meet this challenge of reliable engineering of large biological systems, in the face of unknown complexity. One is to take advantage of increasing automation and experimental throughput to arrive at a functional design through screening large libraries of alternative constructs (Hillson et al., 2019). In order to effectively explore the parameter space, these screens may be guided by techniques, such as directed evolution (Agresti et al., 2010). A more rational approach is to discover designs which are robust to specific uncertainties, as exemplified by control theoretic approaches (Khammash, 2016; Vecchio et al., 2016; Hsiao et al., 2018). In this approach, it is not necessarily required to fully characterize the system, but merely to know which parts of the system are uncharacterized and varying, and therefore need to be buffered by an appropriate architecture.

Finally, a fully bottom-up approach attempts to rationally construct increasingly complex biomolecular systems from basic parts *in vitro* (Liu and Fletcher, 2009; Caschera and Noireaux, 2014a; Göpfrich et al., 2018; Schwille et al., 2018; Ganzinger and Schwille, 2019; Liu, 2019). In this approach, the major interactions within the system can in principle be fully quantified and understood. The payoffs from these efforts are well-informed models and understanding of increasingly complex biological systems (Elowitz and Lim, 2010), which may eventually guide fully predictive design in the future.

The rapidly growing field of cell-free synthetic biology (Garenne and Noireaux, 2019) brought forth numerous examples where such a constructivist approach has been adopted to elucidate basic principles associated with bottom-up construction of biomolecular complexity. The purpose of this review is to give a historical perspective and present an overview of the current capabilities and challenges facing this particular approach. We begin by giving an overview of the rich scientific history of cell-free gene expression systems and their use in deciphering fundamental biological processes by deconstructing them into their essential components. We then describe the current state of bottom-up cell-free synthetic biology, with a dual focus on both the cell-free systems themselves, as well as emerging technological platforms that enable increasingly complex and sophisticated manipulations of cell-free systems. Finally, we discuss how the construction of additional complexity on top of existing TX-TL systems stimulates the investigation of fundamental biological questions, which include context effects in gene expression, resource management, and possibilities for *in vitro* DNA replication.

Reliable engineering of synthetic biomolecular systems is an ambitious goal, whose success will depend on knowledge and insights gained from many different perspectives. We envision that the bottom-up approach, as exemplified in particular by cell-free synthetic biology, will play a key role in enabling the full potential of synthetic biology.

## 2. Deconstructing Biology Using Cell-Free Systems

Cell-free systems are created by extracting cellular machinery, and combining them with energetic substrates and cofactors to recapitulate central biological processes, such as transcription and translation *in vitro*. While this approach has been in existence since Buchner's (1897) observation of cell-free fermentation in yeast extract (Buchner, 1897), it was only during the molecular biology revolution in the 1960s that cell-free systems began to be used in a rational and directed manner to elucidate biological mechanisms.

Early pioneers of cell-free investigations took advantage of two important properties of the system: its simplified biochemical nature, and its open reaction environment. Preparing a cell-free extract strips away much of the complexity of cellular regulation, homeostasis, and growth, revealing the isolated biochemical mechanisms underneath. By reconstituting the basic steps of protein synthesis, *E. coli* cell-free systems were used to demonstrate peptide synthesis from amino acids (Lamborg and Zamecnik, 1960), RNA (Nirenberg and Matthaei, 1961), and finally DNA, via coupled *in vitro* transcription and translation (Wood and Berg, 1962; DeVries and Zubay, 1967; Lederman and Zubay, 1967), thereby experimentally validating the central dogma of molecular biology. The first full protein synthesized *in vitro* was the coliphage F2 coat protein (Nathans et al., 1962).

The open nature of cell-free systems meant that factors which affected protein synthesis could be isolated and characterized, thus allowing direct study of transcriptional and translational regulation. Well-known examples of this work include the direct demonstration of the lac repressor's effect on peptide synthesis (Zubay et al., 1967), and the identification, isolation, and characterization of the catabolite activator protein (CAP) (Zubay et al., 1970). Cell-free systems were subsequently used to identify and elucidate genetic operons in *E. coli* (Zubay, 1973).

Another set of cell-free experiments of fundamental importance was the study of translation from synthetic polyribonucleotides by Nirenberg et al. They observed that cell-free extracts loaded with synthetic poly-uracil led to the production of only one type of polypeptide, poly-phenylalanine (Nirenberg and Leder, 1964). Thus, they hypothesized that poly-U must encode for phenylalanine. Over the next few years, the base composition, triplet nature, and eventually the genetic code mapping DNA sequence to amino acids was determined (Nirenberg et al., 1966).

Over the subsequent few decades, it became a standard approach to use *in vitro* systems to elucidate mechanisms in molecular biology [e.g., RNA replication (Mills et al., 1967), splicing (Kruger et al., 1982), Golgi trafficking (Balch et al., 1984), and chemiosmosis (Steinberg-Yfrach et al., 1998)]. In parallel, the growth of *in vitro* protein synthesis applications drove the development of increasingly efficient cell-free extracts, which achieved greater yields by incorporating more advanced metabolism to energize synthesis and recycle waste products (Jermutus et al., 1998). In the early 2000s, extract engineering merged with the nascent field of synthetic biology, giving rise to the field of cell-free synthetic biology (Noireaux et al., 2003), where instead of reconstituting existing biological processes, novel ones were constructed in the cell-free environment. This synthetic approach continues to characterize the field today.

## 3. Technologies

### 3.1. Lysates and Reconstituted Cell-Free Systems

In recent years the number of cell-free transcription-translation (TX-TL) systems from different organisms has grown rapidly (Zemella et al., 2015; Perez et al., 2016; Gregorio et al., 2019). The most common lysate systems include *E. coli*, insect, yeast, Chinese hamster ovary, rabbit reticulocyte, wheat germ, and human HeLa cells; and newly emerging systems include *B. subtilis* (Kelwick et al., 2016; Yim et al., 2019), *V. natriegens* (Failmezger, 2018; Yim et al., 2019), and *P. putida* (Wang et al., 2018; Yim et al., 2019), among others (Yim et al., 2019). Hybrid systems composed from multiple sources have also recently emerged (Anastasina et al., 2014; Panthu et al., 2018; Yim et al., 2019). Many of these lysate systems are currently commercially available. Concurrent with the expanding set of available lysate systems, there has also been a resurgence of interest in reconstituted recombinant systems, which are composed of mixtures of purified enzyme components. In this review, we will focus on *E. coli* lysate as well as recombinant systems, as they are commonly-used cell-free systems.

#### 3.1.1. *E. coli* Lysates

**Figure 1:**
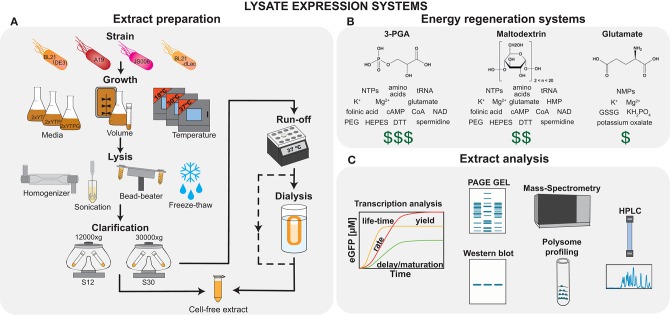
Cell-free lysate systems. **(A)** The major steps in lysate preparation include growth, lysis, and clarification; however there exists a number of variables and options at each step, which can be adjusted to influence the final extract performance. **(B)** Examples of three energy regeneration systems are shown, which offer different cost-performance tradeoffs. **(C)** The final extract composition and performance may be analyzed using techniques, such as protein expression analysis, PAGE gel, Western blot, mass-spectrometry, polysome profiling, and HPLC.

The preparation and performance of *E. coli* lysate-based TX-TL systems vary tremendously and it is well-known that there can be large variability between different batch preparations (Takahashi et al., 2015b). For example, a recent study showed variability of more than 40% for TX-TL systems prepared in different laboratories, which resulted mainly from differences in personnel, and reagents used, and significantly, the laboratory in which the measurement was carried out (Cole et al., 2019). Fortunately, there is an increasing understanding of the role that each of the preparation steps plays in determining the final extract performance, as well as the factors responsible for reproducibility (Silverman et al., 2019b). Proteomics has been applied to elucidate the dependence of lysate composition and performance on batch variability, preparation methods (Failmezger et al., 2017; Foshag et al., 2018), as well as strain variability (Hurst et al., 2017; Garenne et al., 2019). The quest for a deeper understanding is also supported by the use of additional methods, such as metabolomics (Bujara et al., 2011), and other techniques such as polysome profiling (Liu et al., 2005), HPLC (Martin et al., 2018), and gel electrophoresis (Jaroentomeechai et al., 2018) (Figure 1C). These results raise the exciting prospect that lysates will become an engineerable substrate, where standardized and controlled preparation can result in extracts with a variety of defined behaviors. This approach has been particularly powerful in the context of cell-free metabolic engineering, and has been reviewed extensively by Karim et al. (2016) and Karim and Jewett (2018). Here we present an overview of different types of lysate preparation steps (Figure 1A), and their effects on lysate properties. The history of the field, recent advances, as well as the development, optimization, and applications of TX-TL systems are covered in recent reviews (Chiao et al., 2016; Silverman et al., 2019a).

*E. coli* extracts are prepared from a variety of different strains, whose choice strongly depends on the intended application. The most commonly used strains are BL21-derivatives (Sun et al., 2013; Kwon and Jewett, 2015; Didovyk et al., 2017; Cole et al., 2019), but the use of other strains can also be advantageous. For example, strains lacking DNAase, RNAase, and other *E. coli* enzymes can be used to enhance protein yield (Hong et al., 2015; Kwon and Jewett, 2015), for biosensing applications (Didovyk et al., 2017), or for circuit prototyping (Niederholtmeyer et al., 2015).

Different media, such as 2 × YT (Kim et al., 2006), 2 × YTP (Sun et al., 2013; Failmezger et al., 2017), or 2 × YTPG (Kwon and Jewett, 2015), as well as different temperatures and volumes can be used, which will influence the bacterial proteome and thus the composition of the lysate. For example, adding phosphate and glucose has suppressive effects on phosphatase activity (Kim and Choi, 2000). Bacteria can also be harvested at different time points during exponential or stationary phases. Surprisingly, this appears to have very little effect on lysate performance (Kwon and Jewett, 2015; Failmezger et al., 2017).

Cell lysis is a major and variable step of the overall lysate preparation, and different methods result in varying cost, scalability, and ease of use. Bacterial cells can be lysed by sonication (Kwon and Jewett, 2015), high-pressure homogenization (Hong et al., 2015), bead-beating (Sun et al., 2013), or enzymatic auto-lysis (Didovyk et al., 2017). Production yield between systems were shown to be comparable (Sun et al., 2013; Kwon and Jewett, 2015). However, other factors should also be considered. For example, the formation of inverted membrane vesicles is favored in lysates prepared with high-pressure homogenizers, and their preservation is essential for processes, such as oxidative phosphorylation (Jewett et al., 2008) and glycosylation (Jaroentomeechai et al., 2018). Subsequent lysate clarification usually involves centrifugation at 30,000 × g for S30 lysates or 12,000 × g for S12 lysates, which leads to different lysate clarity as distinct components sediment at different speeds, making the S30 lysate less viscous and opaque. For many applications no significant difference was observed between S30 and S12 lysates (Kim et al., 2006); however S12 lysates contain more inverted membrane vesicles which can support oxidative phosphorylation, and hence may be desirable for certain applications.

To reduce preparation time and simplify the process, some steps have been omitted in recent studies. Among these are run-off reaction and/or dialysis (Shrestha et al., 2012; Kwon and Jewett, 2015). Omitting these has minimal influence on final yield in T7 RNAP based systems (Kim et al., 2006; Kwon and Jewett, 2015) and might even be beneficial for retention of co-factors, amino acids, and tRNAs (Calhoun and Swartz, 2005a; Cai et al., 2015). However, the omission of both run-off reaction and dialysis has a profound effect when native transcriptional machinery is used (Kwon and Jewett, 2015; Silverman et al., 2019b).

Another important difference between systems is related to the energy regeneration approaches used (Figure 1B). The first systems based on substrates containing high-energy phosphate bonds (phosphoenolpyruvate, acetyl phosphate, creatine phosphate) were expensive and inefficient because of their fast degradation by non-specific phosphatases, and formation of inhibitory inorganic phosphate molecules. Over the last 20 years, a large amount of work has focused on yield improvement and price reduction. Most current energy regeneration systems are based on the native metabolic pathways of *E. coli*. These use either a part of—PANOx (Caschera and Noireaux, 2014b), 3-PGA (Sun et al., 2013)—or the entire *E. coli* glycolysis pathway—glucose (Calhoun and Swartz, 2005b), maltose (Caschera and Noireaux, 2014b), maltodextrin (Kim and Winfree, 2011; Caschera and Noireaux, 2015), and starch (Kim et al., 2011). These approaches have decreased the price per mg of synthesized protein to under one U.S. dollar. Nevertheless, we still lack systematic studies on the influence of these different energy regeneration methods on lysate properties other than simple protein yield. In particular, for prototyping and characterization of circuits, it is known that resource competition leading to improperly balanced energy usage (Siegal-Gaskins et al., 2014; Koch et al., 2018), efficiency of energy sources and small molecule replenishment (Siegal-Gaskins et al., 2014; Borkowski et al., 2018), changes in binding kinetics due to magnesium ion concentration changes (Kim et al., 2008), and pH variability (Calhoun and Swartz, 2005b) are all dependent on the energy system used and are expected to have profound influence on circuit behavior.

Finally, lysates can be directly supplemented with additives, such as liposomes, polymers, and detergents to facilitate folding of membrane proteins (Hein et al., 2014; Henrich et al., 2015). Enzymes, such as gamS (Sun et al., 2014) or short DNA decoy sequences (Marshall et al., 2017) can be added to prevent linear DNA degradation. The ease of adding functionality to lysates is a major advantage facilitated by the open nature of cell-free reactions.

#### 3.1.2. Recombinant Systems

Lysate systems contain essentially all cytoplasmic components, which is advantageous for recapitulating cellular processes. However, this makes their composition ill-defined, leading to challenges in basic science and engineering. To address these difficulties, efforts were made to generate fully recombinant cell-free systems from a small number of purified enzyme components, whose composition can be defined exactly. Such defined systems are especially important for bottom-up synthetic biology for three main reasons. The first is that their use supports research into minimal cellular systems, as “minimality” of components and pathways can be directly tested. Secondly, the composition of the recombinant system is known much more precisely than for extract-based systems. This property is highly beneficial for modeling, optimization, troubleshooting, and mechanistic understanding of engineered pathways. Thirdly, the use of recombinant cell-free systems presents a viable approach toward the development of *de-novo* constructed synthetic cells.

**Figure 2:**
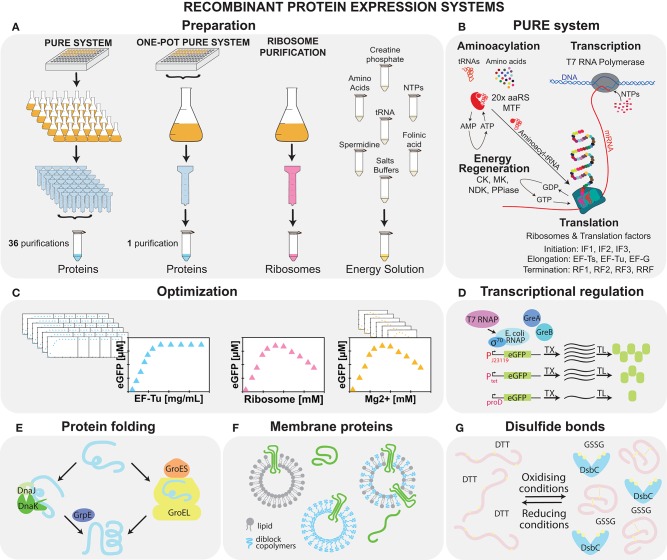
Recombinant cell-free systems. **(A)** Schematic of the preparation of the three elements constituting the PURE system: proteins, ribosomes, and energy solution. **(B)** The four major reactions, aminoacylation, transcription, translation, and energy regeneration occurring during cell-free protein synthesis in the PURE system are shown along with a list of the components involved. **(C)** Optimization of the system can be carried out by adjusting both protein and energy solution components. Potential system modifications are shown: **(D)** supplementation with *E. coli* RNAP allows for more complex transcription regulation (Maddalena et al., 2016); **(E)** addition of chaperones aids protein folding (Niwa et al., 2012); **(F)** vesicles enable membrane protein folding and assembly (Kuruma and Ueda, 2015; Niwa et al., 2015a; Jacobs et al., 2019); and **(G)** oxidizing conditions allow for disulfide bond formation (Shimizu et al., 2005).

Almost half a century ago, Weissbach's group developed the first such systems from recombinant *E. coli* proteins (Kung et al., 1977), but observed very low protein yield. About 25 years later, thanks to the advent of His-tag purification as well as the addition of a creatine-phosphate-based energy regeneration system, Shimizu et al. (2001) developed a very similar system called PURE (protein synthesis using recombinant elements) but with markedly higher protein synthesis yield (Figures 2A,B). Currently, there are three commercially available versions of this system: PUREfrex 2.0 (GeneFrontier), PURExpress (NEB) (Tuckey et al., 2014), and Magic PURE system (Creative Biolabs). Although highly popular, these systems are more expensive (*$*0.6–2/μL) than lysate systems (*$*0.3–0.5/μL). Moreover, despite the fact that the commercial systems are all based on the original PURE system, their exact composition is proprietary, and functional differences can be observed between them in terms of batch to batch variability, system yield, translation rate, lifespan of the reaction, and shelf-life (Doerr et al., 2019).

Cost-effective and modular PURE systems with user-defined compositions can be prepared in the laboratory (Shimizu and Ueda, 2010; Horiya et al., 2017), but the labor-intensive protocol requires ~36 medium to large scale His-tag and ribosome purification steps (Figure 2A). Thus, different approaches to simplify the protocol have been developed, including His-tagging of *in vivo* enzyme pathways (Wang et al., 2012), microbial consortia (Villarreal et al., 2018), and bacterial artificial chromosomes (Shepherd et al., 2017). The first two systems achieved a 10–20% protein yield compared to the commercial PURExpress (NEB). Although the third approach reached protein synthesis levels comparable to PUREfrex, in all three of these approaches it is not possible to rapidly modify protein levels or omit proteins. We recently demonstrated that all proteins, except ribosomes, can be prepared from individual strains in a single co-culture and purification step called the OnePot PURE system, which achieves a similar protein synthesis yield as commercial PURExpress (Lavickova and Maerkl, 2019) (Figure 2A).

Much work has been carried out to improve existing recombinant systems, particularly focusing on the protein expression yield: in addition to increasing the versatility of the system, this has also resulted in a better understanding of the system itself. Improved yield, lower cost, and the ability to adjust the system composition opens up many possibilities for applications, such as the development of defined artificial cells, gene network engineering, biosensors, and protein engineering. Here we separated the various approaches into two distinct types: the first includes experimental and theoretical approaches which aim to find an optimal composition of the system, while the second involves supplementing the existing system with factors that augment its behavior.

One direction for optimizing recombinant systems for protein synthesis yield is focused on finding optimal concentrations of the basic system components, such as proteins, energy sources, small molecules, and salts (Kazuta et al., 2014; Li et al., 2014, 2017; Doerr et al., 2019) (Figure 2C). Important work to improve our understanding of the system was done by Matsuura et al. (2009), who performed titrations of all protein components. These studies showed that although the system is composed of a relatively small number of components, its behavior is complex, and its analysis requires multivariate optimization. One of the most important parameters in the system is the magnesium ion concentration, which influences ribosome function. It is difficult to control the concentration of magnesium ions as they can be chelated by negatively charged molecules, such as NTPs, creatine phosphates, and pyrophosphates (Li et al., 2014, 2017). Studies focused on protein component concentrations showed that the performance of the system is mostly influenced by the concentration of ribosomes and translation factors. Increased yield depended strongly on high concentrations of EF-Tu, which often forms more than 50% of the non-ribosomal protein content *in vivo*. Moreover, finding optimal concentrations is essential for release factors and initiation factors, as an inhibitory effect was shown for these components when higher-than-optimal concentrations were used (Matsuura et al., 2009; Kazuta et al., 2014; Li et al., 2014). Finally, the optimal composition of the system will vary depending on the application. As an example, high concentrations of components, such as NTPs enhance transcription and translation, while inhibiting DNA replication (Sakatani et al., 2015).

To better understand the system behavior and to identify limiting factors, computational models of the PURE system have been developed. This includes coarse-grained ordinary differential equation (ODE) models containing effective lumped parameters and a small number of reactions (Mavelli et al., 2015; Carrara et al., 2018; Doerr et al., 2019), as well as more complex models based on modeling of a large number of elementary reactions, which can provide more detailed mechanistic insights but whose connection to experimental data as well as parameter inference is challenging (Matsuura et al., 2017, 2018). These models show that a number of steps involving ribosomes could potentially become rate-limiting: these include slow elongation rates, peptide release, and ribosome dissociation; qualitatively similar results were observed experimentally (Kempf et al., 2017; Li et al., 2017; Doerr et al., 2019).

As in the case of lysates, a second approach is based on augmenting the system with additional components, such as proteins (Kazuta et al., 2008), crowding agents, and liposomes. For example, yields can be slightly increased by adding proteins, such as EF-4 (Li et al., 2014), EF-P (Li et al., 2017), Pth (Kazuta et al., 2014), and HrpA (Kazuta et al., 2008). Recently, an energy regeneration system originally based on three kinases was replaced by one featuring a single polyphosphate kinase. This improvement lowers the price of the energy source and simplifies the energy regeneration process (Wang et al., 2019). While the original PURE system only contains T7 RNA polymerase, with its limited capability for transcriptional regulation, *E. coli* σ-factor based transcription has been successfully demonstrated, albeit with low efficiency with certain promoters, which can be enhanced by adding purified *E. coli* polymerase alone or in combination with transcription elongation factors (Maddalena et al., 2016) (Figure 2D).

Protein folding can be improved by incorporating chaperones, such as a trigger factor, DnaK/DnaJ/GrpE, and chaperonin GroEL/GroES (Figure 2E). Likewise, Niwa et al. (2012) showed that the solubility of 800 aggregation-prone *E. coli* cytoplasmic proteins can be enhanced if chaperones are added. Furthermore, an oxidizing environment and a disulfide bond isomerase are essential for the expression of proteins containing disulfide bonds (Shimizu et al., 2005) (Figure 2G). The addition of liposomes (Kuruma and Ueda, 2015; Niwa et al., 2015a) together with diblock copolymers (Jacobs et al., 2019) is important for membrane-protein synthesis (Figure 2F). Finally, the concentration of components in the cell-free system is up to 100 times lower than the native *E. coli* cytoplasm. Crowding agents, such as bovine serum albumin (BSA) (Li et al., 2014), Ficoll (Ge et al., 2011), polyethylene glycol (PEG) (Ge et al., 2011; Li et al., 2014), or osmolites (Moriizumi et al., 2019) can help mimic the *E. coli* cytosol (Ge et al., 2011), but they affect both transcription, translation (Norred et al., 2018), and the final synthesized proteins (Niwa et al., 2015b) in a complex way. Further studies will be needed to decipher the various physico-chemical effects of crowding on gene expression. Lastly, it was shown that temperature optimization is a key factor for chaperone-free assembly of protein complexes, such as DNA polymerase (Fujiwara et al., 2013).

### 3.2. Microfluidic Platforms

While cell-free reactions can be carried out successfully in a simple test tube, the complexity and sophistication of experiments can be dramatically augmented by coupling them to the appropriate technological platform. There have been numerous technological advancements with respect to cell-free gene expression over the past few decades, leveraging advances in microarraying, automation, and in particular, microfluidics. Offering reductions of orders of magnitude in sample volume, concomitant low cost, small device footprint, quantitative detection methods, and precise sample manipulation, microfluidic technology has offered tremendous improvements in control and throughput of cell-free reactions (Damiati et al., 2018; Dubuc et al., 2019). We will focus on recent platforms enabling increased control over batch and, importantly, steady-state reactions, as well as describe recent work in the area of compartmentalization.

#### 3.2.1. Increased Throughput and Spatial Control of Batch Reactions

Early high-throughput methods of spatially confined cell-free batch reactions were applied to the generation of protein arrays. In 2004, Ramachandran et al. showed that a plasmid array spotted on a glass slide could be transformed into a protein array by submersing the entire slide in a cell-free reaction. mRNA and proteins were locally transcribed and translated from the spotted plasmid DNA and proximally captured by surface bound antibodies (Ramachandran et al., 2004, 2008). The *in situ* generated protein array could then be interrogated with a protein of interest. A similar concept was later integrated into a microfluidic device for the automated mapping of protein-protein interactions (Gerber et al., 2009). Here linear expression DNA templates are spotted on a glass slide in pairs. The DNA array is then aligned to a MITOMI microfluidic device (Maerkl and Quake, 2007) so that each pair of linear templates is enclosed by a reaction chamber. Loading of the device with cell-free reaction solution synthesizes the bait and prey proteins, which are then assayed for interaction using the MITOMI method. A similar approach was used to generate large numbers of defined bHLH (basic helix-loop-helix) transcription factor mutants to assess the evolutionary accessible DNA binding specificity repertoire of these transcription factors (Maerkl and Quake, 2009). Martin et al. (2012) used the method to generate an RNA array for protein-RNA interaction studies. More recently, hundreds of full-length *Drosophila* transcription factors spanning a size range of 37–231 kDa were expressed on-chip using a wheat germ cell-free system (Rockel et al., 2013). Such approaches are becoming appealing for protein engineering, especially with the rapid decrease in synthetic DNA cost. In 2015, we demonstrated that over 400 synthetic zinc-finger transcription factors could be synthesized and characterized *in vitro* using this approach (Blackburn et al., 2015).

**Figure 3:**
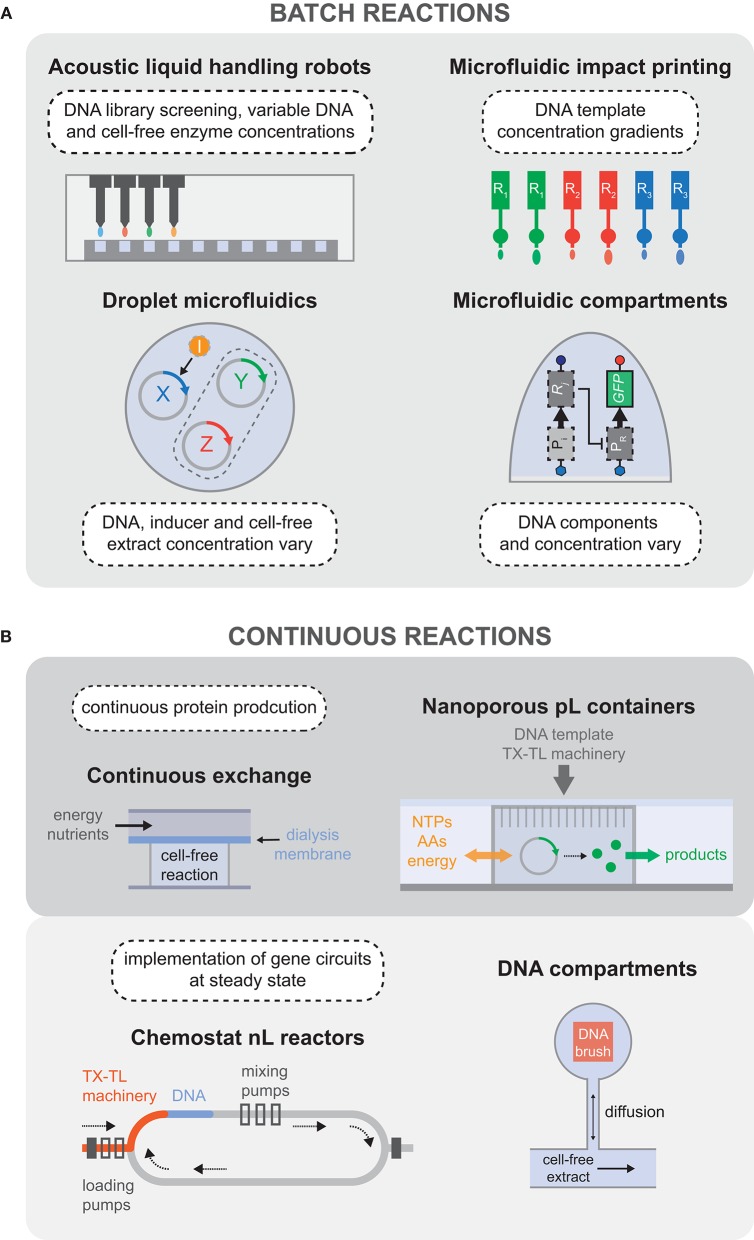
Batch and continuous cell-free reaction platforms. **(A)** Overview of the technologies used to carry out high-throughput batch reactions, including the possibilities to vary the concentration of many reaction components in addition to exploring the sequence space of DNA templates. **(B)** Devices developed for continuous cell-free reactions, separated into two categories: continuous protein production, and steady-state reactors that enabled the implementation of genetic oscillatory circuits.

As synthetic gene networks began to emerge, the advantages of cell-free protein expression were adopted to rapidly screen large libraries of functional DNA parts, avoiding *in vivo* cloning steps, and speeding up the design-build-test cycle (Siegal-Gaskins et al., 2014; Takahashi et al., 2015a). The advent of acoustic liquid handling robots has enabled cell-free reactions to be carried out in standard microwell plate systems with increased throughput and precision, while simultaneously reducing reagent usage. This was recently demonstrated and coupled with a Bayesian modeling approach, which offered a fast route to characterizing regulatory elements from a non-model microbial host (Moore et al., 2018). With their rapid and automated method the authors were able to infer previously unknown transcription factor binding affinities as well as quantify resource competition in cell-free reactions (Figure 3A). Cell-free systems are particularly amenable to mechanistic modeling, and Bayesian inference of model parameters, which benefits from the possibility to perturb the composition of open cell-free reactions. Bayesian approaches uses probability distributions to quantify the degree of belief and uncertainty in the model, and can be deployed to quantitatively compare a number of models as well as determining parameter uncertainty. Automated acoustic liquid handling was also used to test serine integrase recombination dynamics (Swaminathan et al., 2017). A Python package built to model and simulate biological circuits was then applied to the cell-free prototyping data to carry out Bayesian parameter inference.

Microfluidic platforms applied to cell-free TX-TL have also enabled the exploration of larger design spaces at faster time scales. For example, droplet microfluidics was used to rapidly generate a library of distinct combinations of DNA templates, inducer molecules, and cell-free extract concentrations, with the possibility of generating millions of parameter combinations per hour (Hori et al., 2017). Together with a dye labeling scheme, it was possible to create a detailed map of biocircuit expression vs. parameter combination (Figure 3A). Sharing a common goal of characterizing gene network parameters, an alternative microfluidic platform was developed to carry out cell-free TX-TL in high-throughput, using different combinations of surface immobilized DNA as the reaction templates (Swank et al., 2019). Functional repression assays and quantitative affinity measurements (Maerkl and Quake, 2007) were used to characterize a library of synthetic transcription factors, enabling gene regulatory networks to be built from purely synthetic parts *de novo* (Figure 3A). Another quantitative and multi-dimensional study of genetic promoters was carried out using parallel piezoelectric cantilever beams that were able to generate an array of droplets containing cell-free TX-TL reaction mixtures with highly accurate concentration gradients (Fan et al., 2017) (Figure 3A).

Setting aside high-throughput techniques, there exist many other innovative technologies for cell-free gene expression, including methods that have sought to introduce spatial organization. In particular, a chip was developed to separate transcription and translation into different compartments (Georgi et al., 2016). Multi-compartment vesicles were used to predefine regions in which different proteins would be synthesized *in vitro* (Elani et al., 2014). Furthermore, Jiao et al. (2018) fabricated a microfluidic device for the encapsulation of plasmid integrated clay microgels. The incorporation of magnetic beads in the microgels permitted their recovery and re-use in subsequent cell-free TX-TL reactions. A bead-based approach was also used to express and capture recombinant proteins in a hydrogel matrix (Lee et al., 2012). Lastly, surface-bound DNA microarrays were aligned with a hydrogel matrix embedding protein synthesis machinery enabling localized protein synthesis (Byun et al., 2013). These studies will be discussed in more detail in section 3.3.

#### 3.2.2. Steady-State Cell-Free Reactions

While cell-free batch reactions provide a means to characterize gene circuits, parts, and devices, the complexity of biological networks that can be implemented is constrained as the systems quickly reach chemical equilibrium. As discussed in section 3.1.1, batch cell-free reactions quickly equilibrate or reach a state of non-productivity for a number of reasons, such as byproduct or cofactor accumulation and subsequent drift from the initial reaction composition (e.g., inorganic phosphate, Mg^2+^, H^+^), denaturation or degradation of protein components, and simple exhaustion of substrate molecules. This has motivated the development of *in vitro* systems that can exchange reagents over time, maintaining the reaction in a non-equilibrium steady state, and mimicking the dilution and regeneration of cellular components during cell growth. Over 30 years ago there was interest in prolonging cell-free TX-TL reactions by providing a continuous flow of amino acids and energy sources to a reaction chamber from which synthesized proteins and by-products could be removed across an ultrafiltration membrane (Spirin et al., 1988). Successive work aimed to improve protein synthesis yield for cell-free TX-TL reactions by using a dialysis membrane to separate the reaction from the feeding solution of amino acids and energy sources, leading to a semi-continuous reaction (Kim and Choi, 1996; Madin et al., 2000). This idea was then extended to be compatible with standard micro-well plate systems that could be used for higher throughput applications (Mei et al., 2006, 2007; Khnouf et al., 2009, 2010). Following upon the same principles of continuous exchange cell-free reactions, a passive PDMS microreactor was built which separated the feeding and reaction chambers with a dialysis membrane, enabling protein synthesis for up to 15 h (Hahn et al., 2007) (Figure 3B).

Recent improvements in implementing continuous cell-free TX-TL reactions came in the form of novel microfluidic devices. For instance, continuous protein synthesis was demonstrated in an array of cell-sized nanoporous silicon containers that could exchange energy components and materials with the surrounding microfluidic environment (Siuti et al., 2011). In 2013, Niederholtmeyer et al. reported a two-layer PDMS device with eight independent nano-reactors that exchanged reagents at dilution rates similar to those of growing bacteria. Using this device, steady-state TX-TL reactions could be maintained for up to 30 h, enabling the first *in vitro* implementation of genetic oscillator circuits (Niederholtmeyer et al., 2013; van der Linden et al., 2019) (Figure 3B). Using the same device, Yelleswarapu et al. (2018) recently demonstrated the construction of synthetic oscillating networks using sigma-factor-based regulation of native RNAP in *E. coli* lysate. In 2014, Karzbrun et al. demonstrated two-dimensional DNA compartments capable of creating oscillating protein expression patterns and protein gradients. Each DNA compartment was linked to a supply channel by a small capillary channel for continuous diffusion of nutrients and products into and out of the compartment (Karzbrun et al., 2014) (Figure 3B). The geometry of the compartments determined the dilution rate of the reaction, giving rise to different observed reaction kinetics. Using high frequency localized electric field gradients, the same group was able to push the TX-TL machinery away from the DNA brush, thereby arresting transcription and translation. They showed that different biomolecules can be manipulated efficiently depending on the applied voltage and obtained sustained oscillation of gene expression from controlled ON/OFF switching of the TX-TL reaction (Efrat et al., 2018).

### 3.3. Compartmentalized Cell-Free Reactions

Compartmentalizing cell-free reactions spatially segregates a bulk reaction into smaller units. In addition to being a fundamental requirement in the construction of artificial cells, compartmentalized TX-TL opens up a number of scientific and practical opportunities, such as increased throughput for screening, *in vitro* directed evolution, distributed computation, and programmable communication. As discussed in sections 3.2.1 and 3.2.2, microwell plates with reaction volumes as low as 0.5 μL (Marshall et al., 2018), and microfluidic devices with volumes down to femtoliters (Karig et al., 2013), have been used to compartmentalize cell-free reactions.

Below, we will cover different types of compartmentalization including emulsions that allow for the rapid generation of multiple small volume compartments; liquid-liquid phase separation which can recapitulate naturally occurring crowded environments; hydrogels of natural or synthetic origin that immobilize DNA or proteinaceous factors and similarly provide a favorable crowded environment; liposomes which can provide a good starting point in the bottom-up assembly of synthetic cells by encapsulating a gene expression system; and other membrane-enclosed compartments with shells composed of polymers or protein-based materials that will expand the repertoire of physicochemical properties and functionalities.

#### 3.3.1. Emulsion-Based Compartments

Emulsion-based compartmentalization allows for the rapid production of reaction vessels with volumes as low as femtoliters (Shojaeian et al., 2019). *In vitro* compartmentalization of TX-TL was first described in the context of *in vitro* evolution when Tawfik and Griffiths (1998) encapsulated a TX-TL system together with a DNA library of genes coding for an enzyme. Single copies of DNA templates were compartmentalized in ~2 μm aqueous droplets dispersed in mineral oil, creating the crucial genotype-phenotype linkage (Contreras-Llano and Tan, 2018) which is required for selection and enrichment of improved enzymes. This eventually allowed a complete cycle of directed evolution of phosphotriesterases to be carried out (Griffiths and Tawfik, 2003).

**Figure 4:**
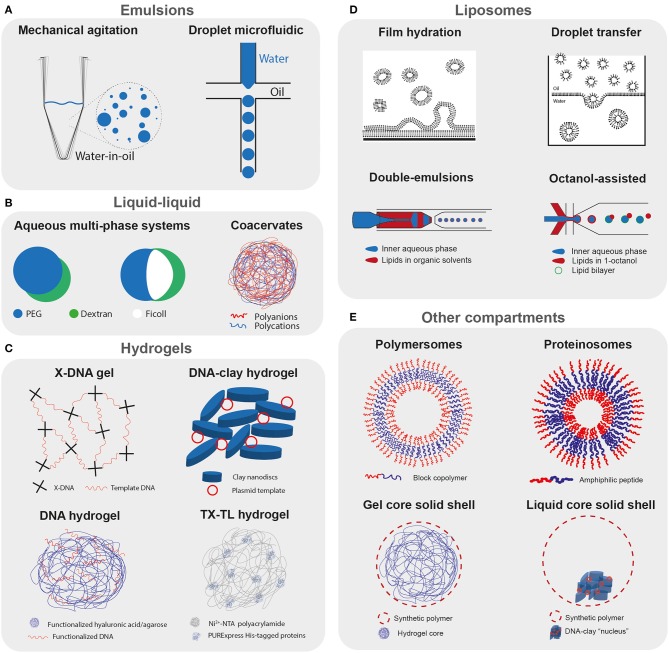
Compartmentalized cell-free reactions. Schematic representation of the different strategies used to compartmentalize cell-free transcription translation reactions. **(A)** Emulsion-based compartments: polydisperse water-in-oil droplets obtained by mechanical agitation, and microfluidic production of monodisperse droplets. **(B)** Liquid-liquid phase separation: aqueous multiphase systems containing cell-free transcription translation machinery (Torre et al., 2014), and representation of a complex coacervate. **(C)** Hydrogels: X-DNA linking template DNA and forming a DNA hydrogel (Park et al., 2009a,b), a DNA-clay hydrogel (Yang et al., 2013), hyaluronic acid (Thiele et al., 2014), or agarose (Aufinger and Simmel, 2018) functionalized with DNA template, polyacrylamide hydrogel functionalized with Ni^2+^-NTA binding PURExpress His-tagged proteins (Zhou et al., 2018). **(D)** Liposomes: rehydration of lipid films with an aqueous solution containing TX-TL, droplet transfer method where a lipid-stabilized W/O emulsion is layered on top of a feeding buffer and liposomes transferred to the bottom by centrifugation (Noireaux and Libchaber, 2004), double-emulsions with ultrathin shells containing lipids in organic solvent (Ho et al., 2015, 2017), and octanol-assisted assembly (Deshpande et al., 2016; Deshpande and Dekker, 2018). **(E)** Other compartments: polymersomes with membrane formed by amphiphilic polymers, proteinosomes with amphiphilic peptides (Vogele et al., 2018), alginate hydrogel coated with various polymers, artificial cells with polymeric shell and liquid core containing a DNA-clay “nucleus” (Niederholtmeyer et al., 2018).

One major drawback of emulsions produced by bulk methods is the size polydispersity of the obtained compartments (Figure 4A). This leads to enzymatic activity being convolved with noise resulting from variation in droplet size, making it difficult to select droplets containing improved enzymes. Dittrich et al. overcame this limitation using droplet microfluidics to generate monodispersed water-in-oil (W/O) droplets (Figure 4A) containing a TX-TL reaction expressing GFP. However, their setup did not allow for the production of droplets containing single DNA copies that gave rise to detectable signals, as would be required for *in vitro* evolution. Using a more efficient TX-TL system and stabilized W/O droplets, Courtois et al. (2008) were able to obtain efficient transcription and translation from a single DNA copy, opening the door for high throughput quantitative evolution experiments in droplets generated by microfluidics. Examples of these include multiple screening rounds to enrich for active hydrogenase (Stapleton and Swartz, 2010) and beta-galactosidase enzymes (Fallah-Araghi et al., 2012).

The use of fluorogenic substrates in enzymatic assays can be problematic in surfactant stabilized emulsions as transport of fluorophores can occur between droplets both in single (Gruner et al., 2019) and double emulsions (Etienne et al., 2018). Woronoff et al. (2015) demonstrated an alternative methodology where a proteinogenic amino acid is released after enzymatic turnover and then incorporated in the translation of a reporter protein. Using this approach, they were able to screen for active penicillin acylase enzymes in single gene droplets. The literature contains fewer examples of compartmentalized *in vitro* assays to screen for protein binders. However, two-hybrid and three-hybrid systems have been developed in PURExpress supplemented with *E. coli* core RNAP enzyme (Zhou et al., 2014). Cui et al. (2016) used such an *in vitro* two-hybrid system encapsulated in single-emulsion droplets to screen a library of 105 peptide binders in a single day.

Recent work using droplets has diversified beyond the high-throughput screening studies discussed in the previous paragraphs to encompass physical effects, such as the influence of crowding (Hansen et al., 2015) or droplet size (Kato et al., 2012; Matsuura et al., 2012; Sakamoto et al., 2018) on protein expression. Schwarz-Schilling et al. (2018) used W/O droplets to compartmentalize streptavidin-coated magnetic beads which act as a scaffold on which complex RNA-protein nanostructures can be built using TX-TL. The high-throughput generation of such compartments is also attractive for the extensive parameter space mapping for genetic network prototyping, as exemplified by the work of Hori et al. (2017) discussed in section 3.2.1.

#### 3.3.2. Liquid-Liquid Phase Separation

Liquid-liquid phase separation occurs when a water-soluble molecule, generally a polymer, is mixed with another aqueous solution containing either a high salt concentration or another water-soluble polymer. Under certain conditions, the first polymer cannot dissolve in the second solution, and a separation into two distinct phases occurs. The resulting “aqueous two-phase system” (ATPS) can form microscale, membrane-less compartments. The recent discovery that ATPS are ubiquitous in cells has attracted much attention to better understand their role in cell physiology (Alberti et al., 2019). Recreating cell-free transcription-translation reactions in these systems could help elucidate the properties of such condensates.

Torre et al. (2014) prepared ATPS of dextran/poly(ethylene glycol) or three-phase systems (A3PS) of dextran/poly(ethylene glycol)/ficoll containing TX-TL by vortexing in mineral oil (Figure 4B). In the ATPS, expression of the reporter protein indicated preferential partitioning of the TX-TL machinery to the dextran phase in the ATPS. The A3PS, on the other hand, exhibited lower expression, which was attributed to separation of TX-TL machinery into the different dextran and Ficoll phases, suggesting that different liquid phases could differentially partition TX-TL components.

When a liquid-liquid phase separated compartment consists of a condensate of biological polymers, it is most commonly referred to as a coacervate (Figure 4B). These coacervates are characterized by a high degree of macromolecular crowding, exhibiting protein concentrations of up to 272 g/L (Deng et al., 2018), similar to the *E. coli* cytosol. Such crowding can profoundly influence gene expression. Sokolova et al. (2013) used a microfluidic device to osmotically concentrate droplets containing lysate, and observed the formation of coacervates in lysate containing 2% PEG-8000. The resultant reporter gene expression was higher in coacervates than in single phase droplets. The work demonstrated that transcription rates were enhanced in the crowded environment of coacervates, offsetting the lower translation rate. Such observations are in agreement with previous studies in bulk cell-free reactions where macromolecular crowding enhances transcription and impairs translation (Ge et al., 2011). To generate monodisperse coacervates in high throughput, Tang et al. (2015) produced coacervates using a microfluidic device (van Swaay et al., 2015) starting from a mixture of carboxymethyl-dextran/polylysine and TX-TL. However, they observed lower gene expression in coacervates compared to the bulk reaction, with results suggesting charge-induced precipitation of the reporter protein after its production. This again indicates that protein expression is sensitive to the partitioning of the TX-TL machinery and that the charge of the coacervate and crowded environment can have opposite effects on yields.

#### 3.3.3. Hydrogels

Similar environments to coacervates are found in hydrogels, where a highly porous hydrated network provides a crowded environment. Forming gel micropads by cross-linking X-shaped DNA entrapping plasmid DNA, or P-gel, Park et al. (2009a,b) obtained an up to 94-fold increase in protein production compared to a standard batch reaction (Figure 4C). They explained the increase in expression by an enhanced transcription rate due to the higher proximity of gene templates in the crowded DNA gel environment. The P-gel has also been prepared in a microdroplet format (Ruiz et al., 2012) and the microgel format was modified with Ni^2+^-NTA to allow the immobilization of the expressed protein on the surface of the microgel (Kahn et al., 2016).

The same group showed that TX-TL was also increased in the presence of a clay hydrogel, which spontaneously forms when mixing hydrated clay in the presence of an ionic solution (Yang et al., 2013) (Figure 4C). DNA and RNA molecules localize to the clay hydrogel and are protected from enzymatic degradation by nucleases. The clay-DNA hydrogels were also formulated into microgels containing magnetic nanoparticles allowing for multiple successive TX-TL reactions after recovery of the magnetic microgel and refreshing of the TX-TL mixture (Jiao et al., 2018). Finally, clay-DNA microgels have been used as artificial nuclei inside W/O emulsions (Jiao et al., 2018) or inside permeable polymeric capsules (Niederholtmeyer et al., 2018).

Thiele et al. (2014) prepared hyaluronic acid functionalized with DNA template and produced porous hydrogel microparticles, which were further encapsulated in droplets containing TX-TL (Figure 4C). They observed efficient GFP protein expression proportional to the number of encapsulated DNA hydrogel beads, with the fluorescent protein diffusing inside the droplet. By using mRNA molecular beacons, they show that the transcribed mRNA remains trapped in the hyaluronic acid/DNA hydrogel, suggesting that transcription and translation both take place inside the hydrogel.

Aufinger and Simmel (2018) prepared agarose functionalized with alkynes and coupled to azide-modified DNA, and used it to prepare hydrogel-DNA “organelles” (Figure 4C). Transcription organelles contained template DNA coding for mVenus with a toehold switch on the 5′ end of the mRNA, whereas the translation organelles were functionalized with the corresponding toehold trigger. These organelles were re-encapsulated in W/O droplets containing TX-TL, and mVenus expression was observed only in droplets containing both the transcription and translation organelles. As these organelles can offer spatial organization of complex reactions while providing continuous exchange with the environment, they are useful for building more complex modular systems.

Whereas the previous studies focused on immobilizing the DNA template inside hydrogels, Zhou et al. (2018) immobilized the complete set of PURExpress His-tagged proteins on a polyacrylamide gel functionalized with Ni^2+^-NTA or an anti-His-tag aptamer (Lai et al., 2020) (Figure 4C). The His-tagged proteins, ribosomes, and template plasmids are placed on pre-dried hydrogel particles, which effectively traps the ribosomes and plasmids in the hydrogel network by convection when rehydrated. Sustained gene expression is observed for as long as 11 days when the cell mimics are constantly supplied with fresh feeding buffer.

#### 3.3.4. Liposomes

Liposomes are compartments encapsulated by a lipid bilayer similar to a cell membrane, making them attractive for the encapsulation of cell-free systems. Liposome technology has been recently reviewed by Stano (2019). Early studies used a film hydration method, where the reaction mix rehydrates a dried lipid film to produce liposomes encapsulating TX-TL (Figure 4D). This was deployed to translate peptides (Oberholzer et al., 1999), proteins (Yu et al., 2001; Oberholzer and Luisi, 2002; Nomura et al., 2003), and finally a more complex genetic cascade (Ishikawa et al., 2004). Noireaux and Libchaber (2004) presented a more convenient method of liposome production called droplet transfer, where a lipid stabilized emulsion of the reaction is first formed in oil and then layered on top of the feeding solution (Figure 4D). Liposomal vesicles are subsequently formed by centrifugation. By producing α-hemolysin *in situ*, which assembled to form pores in the liposome membrane, they were able to constantly supply feeding buffer to the encapsulated reaction and increase the duration of expression up to almost 100 h.

An interesting improvement in the lipid film rehydration method was presented by Nourian et al. (2012) where they dried the lipid films on 200 μm glass beads and rehydrated them with PURExpress. This allowed them to use low reaction volumes to produce liposomes in high yield and with high encapsulation efficiency. Moreover, they used phospholipids with shorter acyl chains to produce semi-permeable liposomes and incorporated biotinylated lipids for efficient immobilization of the vesicles on microscope slides.

Droplet microfluidics allows for the generation of double emulsions with ultrathin shells where the middle phase contains dissolved lipids and forms unilamellar vesicles after evaporation of the solvent (Arriaga et al., 2013) (Figure 4D). Ho et al. (2015) used this technology to encapsulate a mammalian cell-free system with very high encapsulation efficiency, and observe expression of GFP in the interior of the vesicles as well as expression and assembly of a trans-membrane protein. However, they observed in a consequent study that the surfactant necessary for double emulsion led to aggregation of the mammalian cell-free system (Ho et al., 2017).

By using triblock copolymer surfactants, Deng et al. (2016) could control the dewetting of the inner water drop from the middle organic phase thus forming perfectly unilamellar and uniform liposomes, in addition to solvent droplets that could be easily separated. A hierarchical assembly of liposomes inside other liposomes, or vesosomes, through multiple successive encapsulation and dewetting was also demonstrated (Deng et al., 2017). *In vitro* transcription of Spinach RNA was carried out in the interior “nucleus” liposome and translation of mRFP in the surrounding “cytoplasm” liposome, showing great potential toward bottom-up assembly of complex biomolecular structures, even though controlled transfer of mRNA from the interior to the surrounding liposome remains to be implemented. Finally, a similar method called octanol-assisted liposome assembly (OLA) was developed where the middle phase alkane solvents are replaced by octanol containing lipids and undergo rapid dewetting, which could further increase the efficiency and biocompatibility of the encapsulation method (Deshpande et al., 2016; Deshpande and Dekker, 2018) (Figure 4D).

#### 3.3.5. Other Membrane Compartments

Other types of membrane compartments have also been used for cell-free protein expression, such as polymersomes, protein-based membranes, and polymeric shells (Figure 4E). Although there exist many different strategies and materials to make capsules (Cuomo et al., 2019), the conditions necessary for their production often prevent encapsulating cell-free systems. Martino et al. (2012) used a microfluidic capillary device to generate template double-emulsion for the direct encapsulation of a cell-free expression system inside polymersomes composed of PEG-*b*-PLA copolymer and PLA homopolymer to increase their stability. They successfully expressed an MreB protein which formed patches inside the aqueous core and also adhered to the membrane.

Vogele et al. (2018) used a film rehydration method similar to the one used for liposome production but with amphiphilic elastin-like peptides as building blocks, which formed vesicles upon rehydration with a TX-TL system (Figure 4E). They demonstrate that the expression of the elastin-like peptide led to its successful integration into the membrane and an increase in the size of the vesicles after a few hours of expression. Schreiber et al. (2019) also used amphiphilic peptides to form vesicles and encapsulate a cell-free expression system, and show the production and incorporation of amphiphilic peptide in the membrane. It will be interesting to see in future studies if pore-forming proteins can be incorporated in these “growing” protein-based membranes, which might allow for prolonged and higher protein expression, as was observed for cell-free protein expression in liposomes. By encapsulating a cell-free extract in millimeter-sized alginate beads coated with polycationic chitosan (Kwon et al., 2008), silica (Lim et al., 2009), or polyethyleneimine (Saeki et al., 2014), researchers could show continuous expression of eGFP (Figure 4E). However, the core of the capsules presented in the previous studies is in a gel format and it is difficult to assess how well the capsules perform as no absolute quantification of the protein levels was provided.

To our knowledge, the only example to date where cell-free protein expression was demonstrated in liquid core-solid shell polymeric capsules was by Niederholtmeyer et al. (2018) where they produced porous polyacrylate capsules containing a DNA-clay hydrogel nucleus (Figure 4E). The capsules' pores are large enough to allow access by large macromolecules including ribosomes. Transcription-translation from the template DNA immobilized in the clay-DNA hydrogel “nucleus” can be achieved by immersing the capsules in a cell-free expression system. But, as the shell material leads to adsorption of proteins on the capsule surface and the pores are too large to retain the TX-TL machinery, the direct encapsulation of cell-free systems inside polymeric capsules remains to be demonstrated. Such direct encapsulation in synthetic polymeric capsules would be valuable as they could present attractive properties, such as high mechanical and chemical stability, as well as tunable porosity, based on the type of shell material and the fabrication method used.

#### 3.3.6. Physical Effects of Compartmentalization

The effect of the compartment size and interface composition can have notable effects on gene expression. Initial work in Yomo's group showed that expression in sub-picoliter PDMS compartments severely hampered GFP synthesis, whereas quartz glass microcompartments passivated with amino acids showed expression as high as 41% of the test tube reaction with no dependence on compartment volume in a range from 40 fL to 7 pL (Okano et al., 2012). They later showed that synthesis of β-glucuronidase (GUS) with fourth-order reaction kinetics was favored in smaller compartments while GUS substrate depletion was rapidly occurring, pointing to an ideal compartment volume (Matsuura et al., 2012; Okano et al., 2014).

No size dependence on GFP synthesis was observed in a range from 1 to 100 μm in liposomes composed of a mixture of different phosphatidylcholine (PC) or phosphatidylglycerol (PG) lipids and cholesterol (Nishimura et al., 2012), in contradiction to previous reports where PG had inhibitory effect on protein synthesis (Sunami et al., 2010). In lipid stabilized droplets, the charge of the lipid used could also influence the synthesis rate, but in this case the relatively more negative PG lipid was favored over phosphatidylethanolamine (PE) or PC (Kato et al., 2012). Sakamoto et al. (2018) proposed a model with three regimes where there could be activation, no regulation, or repression at the surface. In droplets stabilized by PC lipids, they observed protein expression that did not scale with the droplet volume R^3^, but with R^4^ for droplets with radii below 17 μm, suggesting surface repression in their system. Other effects could explain variations in fluorescence intensity, such as the exchange of solutes between droplets which is influenced by the composition of the carrier oil, lipid or surfactant, as well as the radius of the droplets (Etienne et al., 2018).

The compartmentalization of biochemical reactions in smaller volumes increases the gene expression stochasticity as only a few molecules are present in each compartment. Hansen et al. (2015) suggest that such randomness can be explained by extrinsic noise, which results from the Poisson distribution of encapsulated reagents of the cell-free system, and intrinsic noise, which results from molecular crowding and other parameters, such as the stochasticity of the gene expression reactions or relative plasmid distributions. They co-encapsulated CFP and YFP plasmids in droplets with varying levels of crowding, and observed an increase in intrinsic noise with increased levels of crowding. Intrinsic noise in gene expression can also arise from the stochastic partitioning as was strikingly observed in liposomes prepared in dilute solutions of transcription-translation system (Stano et al., 2013). A small number of compartments (<0.5%) displayed detectable eGFP gene expression, whereas no expression occurred in free solution raising interesting questions about the mechanism of loading of the solute mixture.

High variability in gene expression was also observed in liposomes prepared in PURE solutions of normal concentration and interestingly gave rise to some compartments displaying particularly high or long lasting gene expression (Blanken et al., 2019). These large variations due to stochastic partitioning are interesting as a mechanism to generate diversity in the population, as recently discussed in a review by Altamura et al. (2018). Understanding and harnessing these physical effects of compartmentalization potentially offers yet another way of controlling cell-free gene expression.

#### 3.3.7. Communication

**Figure 5:**
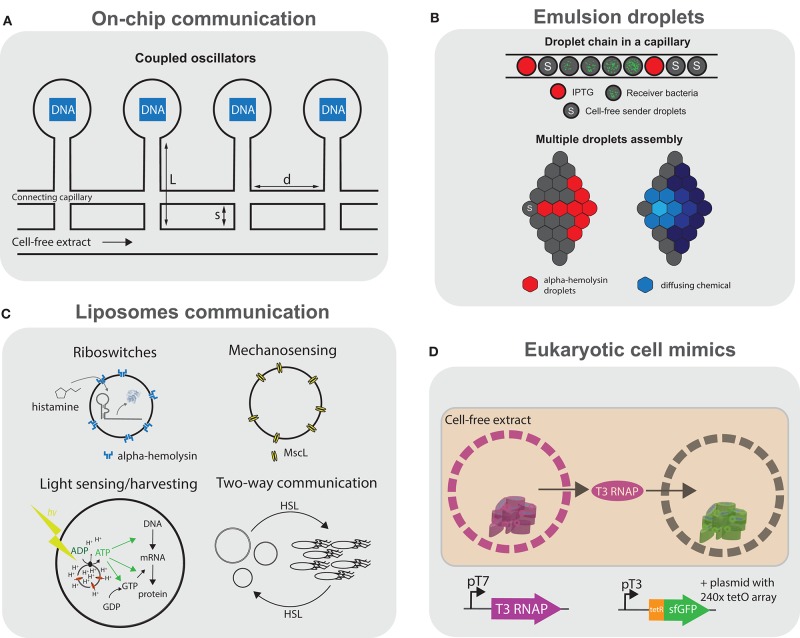
Communication using cell-free transcription translation. Schematic representation of the different platforms using cell-free transcription translation reactions for communication. **(A)** Artificial cells on chip: DNA compartments are connected to a cell-free reaction feeding channel and interconnected by another capillary allowing the coupling of the compartments (Tayar et al., 2015). **(B)** Emulsion droplets: top, water-in-oil droplets containing small molecule activators, bacteria or cell-free genetic circuits arranged in a glass capillary (Schwarz-Schilling et al., 2016); bottom, multiple lipid-stabilized droplets assembled with a micromanipulator with some droplets containing pore forming α-hemolysin (Dupin and Simmel, 2019). **(C)** Sensing and communication with liposomes: liposomes encapsulating histamine-sensitive riboswitches (Dwidar et al., 2019), mechanosensing using MscL pores (Majumder et al., 2017; Garamella et al., 2019), light-driven ATP synthesis using bacteriorhodopsin and ATP synthase (Berhanu et al., 2019), and two-way communication between liposomes and bacteria using various AHLs (Lentini et al., 2017). **(D)** Eukaryotic cell mimics: microporous polymeric capsules containing a DNA-clay hydrogel “nucleus” are immersed in cell-free transcription translation. The expressed T3 polymerase can diffuse and activate transcription-translation in another compartment (Niederholtmeyer et al., 2018).

Cellular communication is fundamental in biology and responsible for many processes ranging from development to tissue homeostasis. Following the successful developments in compartmentalizing cell-free systems, the next logical challenge consists of engineering inter-compartment communication. On-chip artificial cells consisting of DNA brushes (described in section 3.2.2) were interconnected in series by microfluidic channels, and communication is achieved by diffusion of molecules, which can be tuned by adjusting channel geometry (Tayar et al., 2015) (Figure 5A). Diffusion of a σ^28^ activator from one compartment to the next led to sequential switching of a bistable genetic circuit. In a follow-up study, Tayar et al. (2017) used a non-linear activator-repressor oscillator in compartments coupled by diffusion and observed that the oscillators could be synchronized and tuned by geometric control of diffusion. A key demonstration was that such reaction-diffusion systems could spontaneously form spatial patterns in good agreement with theory.

Moving away from microfluidic chips could potentially allow for the engineering of more complex, dynamic consortia of communicating compartments or even tissue-like assemblies. Schwarz-Schilling et al. (2016) used capillaries to align W/O droplets encapsulating cell-free extracts as well as *E. coli* cells (Figure 5B, top). The bacteria and cell-free systems contained either an AND gate circuit expressing GFP in response to isopropyl β-d-1-thiogalactopyranoside (IPTG) and acyl homoserine lactone (AHL), or a sender circuit producing AHL in response to IPTG. Communication could be established between sender droplets and droplets containing the AND gate, in a cell-free-to-bacteria or bacteria-to-cell-free direction.

Dupin and Simmel (2019) used a micromanipulator to arrange multiple directly adjacent W/O droplets in a lipid-in-oil bath, forming a lipid bilayer interface between the compartments (Figure 5B, bottom). They show direct communication between sender droplets containing arabinose (ARA) or AHL and droplets containing a responder circuit. By using an incoherent feed-forward loop genelet circuit containing an RNA binding to 3,5-difluoro-4-hydroxybenzylidene imidazolinone (DFHBI), they observe the propagation of the DFHBI signal along multiple successive interconnected droplets. Finally, by encapsulating a positive feedback circuit expressing α-hemolysin in response to ARA, they observe an increased variability in protein expression levels among droplets, which they describe as “a primitive form of cellular differentiation.”

Liposomes can more closely recapitulate cellular systems. Lentini et al. rehydrated liposomes containing a genetic circuit using a riboswitch responding to theophylline to express α-hemolysin and release co-encapsulated IPTG (Figure 5C). By incubating *E. coli* with these liposomes acting as signal translators, the bacteria could effectively respond to theophylline in the medium (Lentini et al., 2014). They later demonstrated that two-way communication is possible between the artificial cells and bacteria by responding to and secreting different AHLs (Lentini et al., 2017) (Figure 5C). They even devised a “cellular Turing test” where they compare the expression of quorum sensing genes of *V. fischeri* in the presence of either artificial cells or in a consortium of bacteria. They measure that the artificial cells would be 39% “life-like,” but warn that this estimation does not consider that the artificial cells are not fully genetically encoded. Rampioni et al. (2018) developed synthetic cells which could send quorum sensing molecule C4-HSL to the pathogenic *P. aeruginosa*. Such synthetic cells could have interesting theranostic applications once equipped with additional sensing capabilities, such as those discussed in this section.

Two-way communication has been implemented in various contexts, from buffer conditions ideal for artificial cells, to more simple environments, such as water or PBS (Ding et al., 2018). Other communication modalities have also been explored, such as osmoregulation using a mechanosensitive MscL channel incorporated into liposomes, which opens due to membrane stress in hypotonic environments (Majumder et al., 2017; Garamella et al., 2019). Impressively, Berhanu et al. (2019) encapsulated proteoliposomes containing ATP synthase and bacteriorhodopsin inside liposomes (Figure 5C). The artificial cells were able to convert photons to a proton gradient inside the proteoliposomes and drive the synthesis of ATP by ATP synthase, fueling the TX-TL system, effectively making these artificial cells capable of light sensing and even photosynthetic activity.

More complex communication between liposomes was presented by Adamala et al. (2016), where they use artificial cells containing either bacterial or mammalian TX-TL systems and use small molecules to communicate between the prokaryotic and eukaryotic artificial cells containing different genetic circuits and cascades. However, the sensing of small molecules is limited to known transcriptional regulators or the theophylline riboswitch. Dwidar et al. (2019) engineered a riboswitch for the biologically relevant small molecule histamine into liposome-based artificial cells, which could respond to the presence of histamine in a variety of programmed ways (Figure 5C). Finally, liposome-based artificial cells expressing *Pseudomonas* exotoxin A were injected *in vivo* inside mice tumors and an increase in caspase activity was shown (Krinsky et al., 2017), suggesting their potential use in therapeutic or diagnostic applications.

One major limitation of liposomes is the difficulty in implementing signaling mediated by protein factors, as only small signaling molecules can cross the lipid bilayer with the help of the α-hemolysin pore. The polymeric capsules presented by Niederholtmeyer et al. (2018) (as discussed in section 3.3) are permeabilized by 200–300 nm pores, allowing for the exchange of polymerases and even ribosomes (Figure 5D). The authors show a basic form of quorum sensing where the reporter expression increases sharply at a threshold of 400 cell-mimics per 4.5 μL droplet of TX-TL.

Models have been recently proposed to help understand and implement communication using cell-free systems. These include studies of quorum sensing (Shum and Balazs, 2017) and the design of spatially distributed compartments (Menon and Krishnan, 2019). More complex spatial assemblies of compartments capable of communication (Villar et al., 2013), combined with computation by cell-free TX-TL genetic circuits or other *in vitro* computation methods [such as DNA strand displacement reactions (Joesaar et al., 2019), the Polymerase-Exonuclease-Nickase (PEN) DNA toolbox (Genot et al., 2016), or transcriptional “genelet” circuits (Weitz et al., 2014)], and integration with orthogonal technologies, such as electronics (Selberg et al., 2018) may one day allow for the bottom-up engineering of programmable tissues with distributed functional capabilities.

## 4. Scientific Opportunities

The technical achievements described above have given rise to new research directions involving cell-free gene expression systems. While the pioneering scientific applications of cell-free systems have been the deconstruction and elucidation of molecular biological pathways, today the research landscape is much more varied. Of the numerous active research directions (including biosensing; biomanufacturing; diagnostics; screening; minimal, semi-synthetic, synthetic, and artificial cells; education; and genetic, metabolic, and protein engineering), here we highlight three topics which are particularly relevant in the context of bottom-up construction using cell free systems.

### 4.1. Gene Expression Regulation

We still lack a complete appreciation for how cells encode, execute, and regulate gene expression (Phillips et al., 2019), which restricts our ability to predictively design new gene regulatory networks or efficiently compose existing modules. Ever since cell-free systems were used to uncover the central dogma, they have contributed profoundly to our understanding of gene expression (Zubay, 1973). In this line of research, PURE and extract systems bring complementary advantages. The PURE system is based on the core components required by the central dogma, and accordingly, can serve as the foundation from which we can build-to-understand basic aspects of gene expression. Extract-based systems serve as environments more similar to their *in vivo* counterparts, but lacking endogenous mRNA and DNA, effectively decoupling them from host processes that can convolute design implementation and data interpretation (Siegal-Gaskins et al., 2014). This section will highlight recent work that has advanced our understanding of gene expression using cell-free systems to operate at the fertile interface between *in vitro* biochemistry and *in vivo* cell biology.

Biology employs promoters to process input logic and initiate informed transcriptional output (Bintu et al., 2005), an operation believed to lie at the heart of cellular decision-making, yet for which we still possess an incomplete understanding. In investigations of transcriptional regulation, cell-free biology has the benefit of combining complex functional assays with controlled and accessible environments. In contrast to purely *in vitro* research of promoter DNA and transcription factor interactions, cell-free systems have the potential to bridge the divide between promoter occupancy and mRNA production, and help to improve our understanding of the factors that drive transcription. Research from our laboratory by Swank et al. (2019) used cell-free extract to study the interaction between promoters and the largest family of transcription factors, zinc-fingers. They leveraged the compatibility of cell-free systems with high-throughput assays to quantify the binding-energy landscapes of several synthetic zinc-finger regulators (Blackburn et al., 2015). The precise tuning of repression strength was demonstrated, by mutating the consensus sequence or flanking regions to create small changes in binding affinity. This control facilitated the engineering of gene circuits; adjusting individual binding-site affinities was crucial for optimizing logic gate function for example. By fusing interaction domains to repressors, cooperativity was engineered between different regulators binding to promoters possessing two binding sites. With the appropriate placement of binding sites, it was shown that cooperativity greatly increased fold-repression and response non-linearity. Notably, the optimal spacing between cooperative repressors was tied to the helical twist of DNA. The repression strength was greatest if the spacing was such that both repressors would bind to the same face of DNA, while repression decayed to match the non-cooperative level as the spacing changed to place the repressors on opposing sides of the DNA. The combination of predictable cooperative interactions and tunable binding affinity guided the engineering of NAND, AND, and OR gates.

Moving away from intragenic composition, intergenic compositional context effects (referring to the position and orientation of entire genes relative to each other on DNA) have also been shown to influence transcriptional regulation (Rhee et al., 1999; Shearwin et al., 2005; Chong et al., 2014; Yeung et al., 2017). Yeung et al. (2017) arranged genes in convergent, divergent, and tandem orientations, and modeled the relationships (based on torsional stress) between supercoiling and transcription, to support a picture of how supercoiling mediates transcriptional coupling between physically connected genes. Cell-free experimentation served as an important part of the toolkit used to validate their hypotheses and provide evidence for their model. Using cell-free systems, the authors were able to adjust gyrase expression freely, to relax supercoiling and observe the impact on reporter-gene transcription, while avoiding any interference by host-mediated effects. Running cell-free experiments also allowed the authors to control against possible effects coming from plasmid replication. Furthermore, by employing the common practice of expressing linear DNA in cell-free systems (Sun et al., 2014), Yeung et al. were able to investigate the outcome of dissipating peripheral torsional stress, since the ends of linear DNA can rotate freely in response to transcription. Using their insights, the authors leverage supercoiling to build a convergently-oriented toggle switch, which shows a sharper threshold for switching between stable states than the original toggle switch with divergent genes (Gardner et al., 2000).

### 4.2. Resource Constraints as a Design Feature

**Figure 6:**
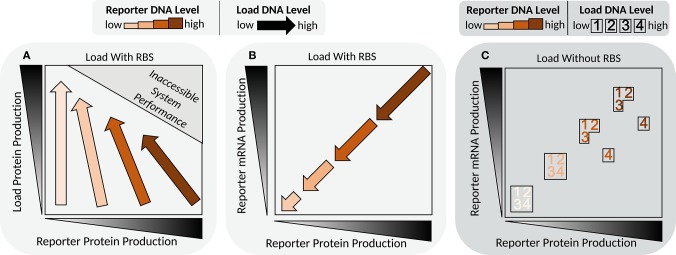
Identifying resource constraints with cell-free gene expression. Schematic summary of results obtained by Siegal-Gaskins et al. (2014) **(A)** The authors observed that at greater reporter DNA concentrations, a given load imposed on the system will produce a larger decrease in reporter protein expression. **(B)** Loading decreased both transcriptional and translational output from cell-free extract. **(C)** When the load DNA lacked a ribosome binding site, loading had no effect, except for at the highest combined load and reporter DNA concentrations, suggesting that the bulk of the imposed load is realized through translational processes. In the figure, the relative positioning of numbers in a given box is arbitrary.

A current focal point in synthetic biology research is understanding the failure of synthetic biomolecular circuitry due to the coupling of individual circuit components through their competition for the same gene expression resource, and the added coupling with host processes seen in *in vivo* implementations (Cardinale and Arkin, 2012; Carbonell-Ballestero et al., 2016; Qian et al., 2017). This category of problems, along with other context dependencies, leads to a reduction in design composability, worsening in proportion to circuit size. In recent years, cell-free systems have served as an important research tool to deepen our understanding of resource constraints. Siegal-Gaskins et al. (2014) exploited the freedom with which DNA concentrations can be varied in cell-free systems to independently quantify the levels of transcriptional and translational cross-talk in cell-free extract (Figure 6). They show that increasing the concentration of a second load construct in their reaction results in a decrease in the transcription and translation of the original reporter construct (Figure 6B). Loading was largely abolished when the second construct lacked a ribosome binding site (Figure 6C), suggesting that the resource bottleneck was caused primarily through increased protein translation. This result was later found to generalize to *E. coli*. (Gyorgy et al., 2015). The effect of an increase in load DNA concentration on reporter protein translation is dependent on the total DNA concentration in the system. At higher total DNA concentrations, translational coupling between genes increases. This was observed experimentally by Siegal-Gaskins et al., where increasing the load DNA in the cell-free system has a greater impact on reporter protein expression when the system contains higher reporter DNA concentrations (Figure 6A). In contrast, the way an increase in load DNA concentration affects transcription was found to be independent of DNA for a larger range of concentration values. This result highlights a limiting translation (but not transcription) capacity, which above a certain level of load, causes a simple resource trade-off between proteins being produced.

A promising direction to improve predictability when composing synthetic parts, in light of resource problems, is to take the primary resources into account in mathematical models, thereby considering non-regulatory interactions between components through resource sequestration (Gyorgy et al., 2015; Gorochowski et al., 2016; Qian et al., 2017). Gyorgy and Murray (2016) developed a model that used the previous cell-free extract data obtained by Siegal-Gaskins et al. to account for resource competition between genes. They were able to successfully predict expression profiles of multiple co-expressed parts, from data where these parts were characterized individually.

Ceroni et al. (2015) developed a “resource capacity monitor” assay implemented in *E. coli*, designed to obtain a measure of load imposed on the host by synthetic circuits. They genomically integrated a GFP gene whose output was used to infer the load imposed by synthetic circuitry, from the relative decrease in GFP when the load is expressed in the host. In a subsequent paper, the same group established a similar approach but using cell-free extract (Borkowski et al., 2018), with the reasoning that this avoids growth-dependencies, which cause results to be difficult to interpret since the burden affects growth rate and promotes mutations. They feed the resource-impact data generated from cell-free experiments into a computational model to estimate the resource cost that would be imposed on cells expressing synthetic circuitry employing the proteins they characterized. This strategy could be integrated with cell-free prototyping workflows, to improve the transfer of circuit design from cell-free to *in vivo*, by creating the opportunity to reject resource-demanding implementations. Furthermore, it is imaginable that cell-free extract systems could be adjusted to be resource-constrained in ways that better emulate a given host in order to improve predictive capacity.

Yelleswarapu et al. (2018) developed a clever oscillator design in cell-free extract that employs resource competition as a functional feature. Their delayed negative feedback topology leverages asymmetric competition between different sigma factors for core RNAP. Studies in this vein can help to improve our understanding of resource competition. By making resource sequestration a design element, circuit failure due to any “cross-talk” through this resource can be reframed as a problem of robust design. By learning design strategies that exhibit the desired behavior over large areas of parameter space, and by figuring out what models properly describe such circuits, we can learn to operate with, and perhaps around, the resource constraints in our biological systems. Even if such a circuit could be implemented successfully *in vivo* using an orthogonal RNAP and sigma-factor system, it would be difficult to untangle the signal of interest from the effects of the asymmetric load that would be imposed on the host. It would be interesting to investigate other resource-related phenomena, like modes of resource coupling or circuit failure following system overloading, using microfluidic chemostats (section 3.2.2), where reaction resources can be varied in a dynamic yet controllable manner.

One interesting strategy to alleviate the resource demands of translation is to implement transcriptional regulation with nucleic-acid hybridization interactions in cell-free systems (Chou and Shih, 2019). Chou et al. were able to do this by functionalizing T7 RNAP with single-stranded DNA, so that it can interact with cis-regulatory ssDNA domains on promoters, in a way that is dependent on nucleic-acid assemblies acting analogously to transcription factors. Although this may not directly advance our understanding of how biology encodes native promoters, making the link between gene regulatory networks and DNA strand-displacement reactions could reduce the cost of scaling up computation in genetic circuits, in order to fast-track the investigation of more sophisticated phenomena.

### 4.3. *In vitro* DNA Replication

Replication and propagation of genetic material is a key feature of life and is distributed among all living systems, and a robust *in vitro* implementation is crucial in particular for efforts in bottom-up construction of synthetic cells. While self-replicating systems including autocatalytic peptides, ribozyme replication, or RNA replicators have been established in the past (Ichihashi, 2019), it is crucial to develop a DNA replication system with regard to a transcription-translation based synthetic cell. Here we will focus on efforts to reconstitute DNA replication processes using cell-free TX-TL.

Organisms have evolved a great variety of mechanisms to replicate their DNA, with a broad range of complexity ranging from the eukaryotic replication machinery [consisting of at least five components some of which are further subdivided into complexes (Berg et al., 2012)], bacterial chromosome and plasmid replication, to simpler bacterial and viral replication strategies. Efforts to achieve *in vitro* reconstitution of DNA replication have focused mostly on the simpler systems.

In the 1980s, researchers reported *in vitro* DNA replication in crude cell extract of infected or transfected cells, including replication of plasmid RSF1010 in *P. aeruginosa* and *E. coli* (Diaz and Staudenbauer, 1982), and SV40 virus in monkey and human cell extract (Li and Kelly, 1984; Stillman and Gluzman, 1985; Wobbe et al., 1985). By the end of the decade, *in vitro* amplification of DNA became routine with the development of the polymerase chain reaction (PCR). Originally using the Klenow fragment of *E. coli* DNA Polymerase I, which was added anew after each hybridization step (Mullis and Faloona, 1987), the PCR method eventually adopted thermostable polymerases enabling continuous thermal cycling. However, repeated thermal cycling is not ideal for future applications involving synthetic cells, and so work on developing isothermal DNA replication methods remains of interest in this context.

Successful reconstitution of these isothermal machineries was eventually achieved *in vitro*, using partially or entirely recombinantly expressed and purified elements. Examples of these include the *E. coli* replication machinery (Kaguni and Kornberg, 1984; Su'etsugu et al., 2017), RSF1010 replication (Scherzinger et al., 1991), and viral replication systems including the phi29 (Blanco et al., 1994), T7 (Hürtgen et al., 2019), T4 (Schaerli et al., 2010), or SV40 (Waga et al., 1994) replication machineries.

**Figure 7:**
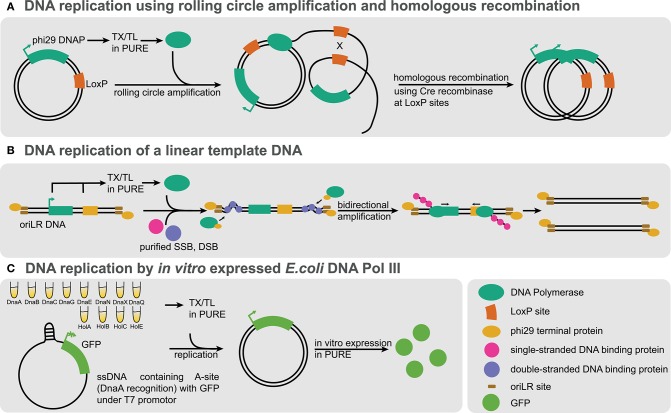
Coupling DNA replication and cell-free gene expression. Schematic representation of methods to couple *in vitro* transcription-translation to DNA replication. **(A)** Sakatani et al. (2015, 2018) coupled *in vitro* expression of phi29 DNAP to rolling circle amplification of circular DNA and finally concluded their round of replication by re-circularizing the replicated DNA using homologous recombination by Cre recombinase at LoxP sites. **(B)** van Nies et al. (2018) reconstituted the native phi29 life cycle by replicating a linear DNA template flanked by oriLR sites expressing phi29 DNAP and TP *in vitro*, and adding recombinant SSB and DSB to the reaction. **(C)** Fujiwara et al. (2013) expressed the *E. coli* DNA Pol III holoenzyme *in vitro*. The enzyme was shown to replicate the second strand of a single stranded linear template containing an A-site; the resulting duplex DNA enables GFP expression.

The establishment of the PURE transcription-translation system has paved the way toward coupling *in vitro* protein expression with DNA replication, with the ultimate aim of reconstituting a self-sustaining system. Sakatani et al. (2015) expressed the phi29 DNA polymerase (DNAP) in PURE from a circular DNA template, which was then able to replicate the latter via a rolling circle amplification. The same group further developed their system based on a concept proposed by Forster and Church (2007), introducing recombinantly expressed Cre recombinase, that re-circularized an evolved form of the DNA template at the lox sites (Sakatani et al., 2018) (Figure 7A). They took advantage of the tunability of their home made PURE system by optimizing the NTP concentration, which is necessary for protein expression, yet was shown to inhibit DNA replication. van Nies et al. (2018) reported that PURE-expressed phi29 DNAP and terminal protein (TP) were able to amplify a linear DNA template encoding both proteins, in presence of recombinantly expressed single stranded and double stranded binding proteins (SSB, DSB) (Figure 7B). Those four proteins were shown to be necessary and sufficient for DNA replication of the phi29 bacteriophage (Blanco et al., 1994; Salas et al., 2016).

Fujiwara et al. implemented an *in vitro* DNA replication machinery by mimicking *E. coli* DNA replication (Figure 7C). Using the PURE system, they expressed the machinery consisting of initiator (DnaA), helicase and helicase loader (DnaB and DnaC), DNA primase (DnaG), and the DNA polymerase III holoenzyme consisting of nine different proteins. By achieving the correct assembly of the holoenzyme in PURE, they furthermore showed the possibility to assemble a complex holoenzyme in the absence of chaperones by decreasing the cell-free expression temperature. The *in vitro*-expressed proteins were able to replicate an artificial gene circuit which expressed GFP in the PURE reaction system (Fujiwara et al., 2013).

Despite these advances, one major challenge on the way to implementing a self-sustaining DNA replication system remains to be addressed. Current approaches couple gene expression with DNA replication using only a couple of consecutive batch reactions. To ensure continuous replication in a future synthetic cell, it will be necessary to achieve continuous, multi-round replication, which could be explored for instance, in microfluidic chemostats as described in section 3.2.2. It has yet to be demonstrated that DNA replication can be achieved over many consecutive cycles, which may prove to be rather challenging as it appears that current DNA replication methods are rather inefficient and produce DNA in low-quantities (Sakatani et al., 2018; van Nies et al., 2018).

During long term replication, mutations will appear, among which some will enable the mutated DNA template to replicate faster than the original template, due to length or altered codon usage. This parasitic DNA may eventually out-compete the original DNA template, if no selection pressure is applied. Compartmentalization, as discussed above in section 3.3, may be a method to address this challenge, as discussed in Ichihashi (2019). Furthermore, implementation of a stable, continuous platform for *in vitro* DNA replication would enable the study of the evolutionary dynamics of molecular replicators, as the system is well-defined, simple, tunable, and does not rely on life-sustaining processes. This may additionally be linked with compartmentalization, where *in vitro* evolution of DNA polymerase using an error prone PCR approach has already been reported (Ghadessy et al., 2001).

*In vitro* coupling of transcription-translation with DNA replication is just at the beginning of its development, and it will be interesting to see what the limitations of the systems are. To our knowledge, only phi29 genomic DNA and plasmids have been replicated using coupled *in vitro* expression/replication systems to date. Successful determination of limits, such as size, accuracy, and energetic requirements to carry out *in vitro* replication may eventually enable the self-replication of all genes required to sustain a synthetic cell.

## 5. Outlook

The bottom-up approach is but one way of addressing the formidable challenge of reliably building complex synthetic biological systems, and it will necessarily be combined with other complementary methods. However, the key principle of building to understand is undoubtedly a powerful motivation, and cell-free systems represent perhaps one of the best examples where this is currently being put into practice. While cell-free systems have historically been used to deconstruct biology, allowing its core processes to be elucidated, recent advances have led to its increasing application to construct biological systems.

Today, basic cell-free lysate systems are less of a black-box, and better characterization of their properties and preparation methods has made them an increasingly engineerable, and maybe more importantly, accessible tool. Recombinant systems have been the focus of increasing investigation as users demand more modularity and cost-effectiveness. Technological innovation in automation, microfluidics, and materials science have enabled increased throughput, dynamic control of steady-state reactions, and sophisticated compartmentalization strategies, while at the same time becoming accessible to more labs around the world.

However, there are also clear challenges ahead. Compartmentalizing cell-free reactions has exposed important physical effects, such as crowding and differential partitioning, which, while complex, may one day be harnessed to control the microscale spatial organization of gene expression. This level of fine control, exhibited by all cells, currently eludes us. Cell-free gene expression studies have unveiled a number of effects, such as physical properties of promoters, supercoiling and compositional context dependencies, and the ever-present resource burden of heterologous gene circuits. Replication studies have pointed out to the difficulty of achieving efficient DNA replication and protein synthesis in a cell-free reaction. And while increasingly complex communication systems have been implemented, the field is still in a nascent stage.

A common theme in constructing complex systems is emergence: as the system grows in size, effects appear which cannot be predicted by assessing the parts independently. In synthetic biology, these confounding effects currently stymie many efforts. But it is exactly because cell-free studies allow us to work at the interface between simple and complex systems that they are well-poised to address these issues. Ultimately, a thorough understanding of these effects will allow us to turn what are currently viewed as design constraints into design features, thereby expanding the scope and potential of synthetic biology.

## Author Contributions

BL wrote section 3.1, ZS wrote section 3.2, GM wrote section 3.3, AS wrote sections 4.1 and 4.2, LG wrote section 4.3. NL wrote sections 1, 2, and 5. SM and NL contributed to all sections. All authors edited the manuscript and approved it for publication.

### Conflict of Interest

The authors declare that the research was conducted in the absence of any commercial or financial relationships that could be construed as a potential conflict of interest.
